# Normal pressure hydrocephalus associated with COVID-19 infection: a case report

**DOI:** 10.1186/s12879-022-07184-x

**Published:** 2022-03-03

**Authors:** Thaís de Maria Frota Vasconcelos, Paulo Ribeiro Nóbrega, Glauber de Menezes Ferreira, Moysés Loiola Ponte de Souza, Alander Sobreira Vanderlei, José Daniel Vieira de Castro, Pedro Braga-Neto, Manoel Alves Sobreira-Neto

**Affiliations:** 1 Hospital Universitário Walter Cantídio, Universidade Federal do Ceará, Fortaleza, Brazil; 2 Department of Clinical Medicine, Neurology Section, Universidade Federal do Ceará, Fortaleza, Brazil; 3 Neurology Service, Hospital Geral de Fortaleza, Fortaleza, Brazil; 4 Neurology and Neurosurgery Service, Hospital Geral Otoclínica, Fortaleza, Brazil; 5 Center of Health Sciences, Universidade Estadual do Ceará, Fortaleza, Brazil; 6 Unichristus University, Fortaleza, Brazil

**Keywords:** Normal pressure hydrocephalus, SARS-CoV-2, Coronavirus, COVID-19, Neurologic manifestations, Case report

## Abstract

**Background:**

COVID-19 is a pandemic disease responsible for many deaths worldwide. Many neurological manifestations have been described. We report a case of normal pressure hydrocephalus (NPH) 2 months after acute COVID19 infection, in a patient without other risk factors.

**Case presentation:**

A 45-year-old male patient presented an 8-month history of progressive gait disorder and cognitive impairment after being hospitalized for SARS-CoV-2 infection. Magnetic resonance imaging (MRI) was compatible with NPH. A spinal tap test was positive and there was progressive improvement after shunting, with complete resolution of symptoms.

**Conclusion:**

Other infections such as syphilis, cryptococcosis and Lyme disease have been associated with NPH. Possible mechanisms for NPH after COVID include disruption of choroid plexus cells by direct viral invasion or as a result of neuroinflammation and cytokine release and hypercoagulability leading to venous congestion and abnormalities of CSF flow. Given the significance of NPH as a cause of reversible dementia, it is important to consider the possibility of a causal association with COVID19 and understand the mechanisms behind this association.

## Background

COVID-19 is a pandemic disease caused by an RNA virus named SARS-CoV-2 and is primarily a respiratory disease involving lower and upper airways, although many neurologic manifestations have been described [1]. Similarly, to what has been reported with other coronaviruses, such as MERS-CoV and SARS-CoV-1, this pathogen has shown possibly neurotropic and neuroinvasive properties resulting in a wide range of neurologic manifestations [1].

Normal pressure hydrocephalus (NPH) is a condition characterized by a triad of cardinal symptoms composed by gait disorder, urinary incontinence and cognitive decline associated with dilated ventricles, in the absence of intracranial hypertension [2].

Herein, we report a case of Normal Pressure Hydrocephalus occurring two months after acute COVID19 infection.

## Case presentation

A 45-year-old man presented with a history of imbalance and progressive gait disorder for the last 8 months, associated with memory impairment. He had a previous history of bipolar disorder and type II diabetes mellitus and reported a COVID19 infection 2 months before initial presentation of neurologic symptoms.

Initial symptoms of SARS-Cov-2 infection were fever, anosmia, dysgeusia and anorexia. A real-time reverse-transcriptase-polymerase-chain-reaction assay (Allplex™ SARS-CoV-2 Assay—N/RdRP/S genes) confirmed the diagnosis. Hospitalization for seven days and oxygen via nasal cannula were necessary. At discharge, he was able to walk unassisted.

Two months after acute COVID19 infection the patient started a progressive gait disorder, complaining that he felt like his feet were glued to the ground. After a few weeks, he started forgetting recent facts and his family complained of excessive daytime sleepiness and blunted affect. He progressed to executive dysfunction and difficulty in performing his labor activities. There was no history of head trauma, subarachnoid hemorrhage (SAH) or meningitis. There was also no family history of neurologic diseases.

Neurologic examination revealed short-term memory loss, slow processing speed, inattention, difficulty in planning and executive dysfunction. A mild tetraparesis was also perceived, with brisk deep tendon reflexes in four limbs. There was no clonus and flexor plantar responses were observed. There was mild rigidity of the upper limbs, without cogwheeling. Gait was slow and magnetic, with short steps, feet close to the ground and postural instability.

Laboratory evaluation did not reveal any abnormalities (Table 1). Complete blood count, renal function, liver enzymes and thyroid hormones were normal. Serology was negative for Syphilis and HIV. Vitamin B12 levels were also normal.

**Table 1:** Laboratory and cerebrospinal fluid analysis in a patient with normal pressure hydrocephalus after COVID19

Variables	COVID symptoms onset	Neurological symptoms onset	Reference values
Serum tests			
Hemoglobin (g/dL)	15.9	14.5	13–17.5
Leukocytes (cells/mm^3^)	7640	5600	4000–11,000
Lymphocytes (cells/mm^3^)	770	2420	1000–3500
Platelets (number/mm^3^)	259.000	255.000	150,000–450,000
C-reative protein (mg/dL)	6.37	–	< 0.3
ESR (mm/h)	27	–	
D-Dimer (ng/mL)	300		< 600
Sodium (mMol/L)	–	142	136–146
Potassium (mMol/L)	–	4,3	3.5–5.1
Calcium (mg/dL)	–	9,3	1.16–1.32
Magnesium (mg/dL)	–	2,3	1.6–2.6
Blood urea nitrogen (mg/dL)	21	14	7–20
Creatinine (mg/dL)	1.1	0.92	0.5–1.3
AST (U/L)	39		
ALT (U/L)	49		
TSH (µlU/mL)	–	1542	0.550–4.780
HIV I, II	–	Negative	Negative
Syphilis	–	Negative	Negative
Analysis of cerebrospinal fluid			
Cell count (cells/mm^3^)	–	2	0–4
Differential cell count	–	100% lymphocytes	–
Protein (mg/dL)	–	26	15–45
Glucose (mg/dL)	–	74	–
VDRL	–	Negative	Negative
Gram stain	–	Negative	Negative
Fungal stain (India-ink)	–	Negative	Negative

*PT* prothrombin time; *aPTT* activated partial thromboplastin time; *VDRL* Venereal Disease Research Laboratory; *PCR* polymerase chain reaction

**Fig. 1:**
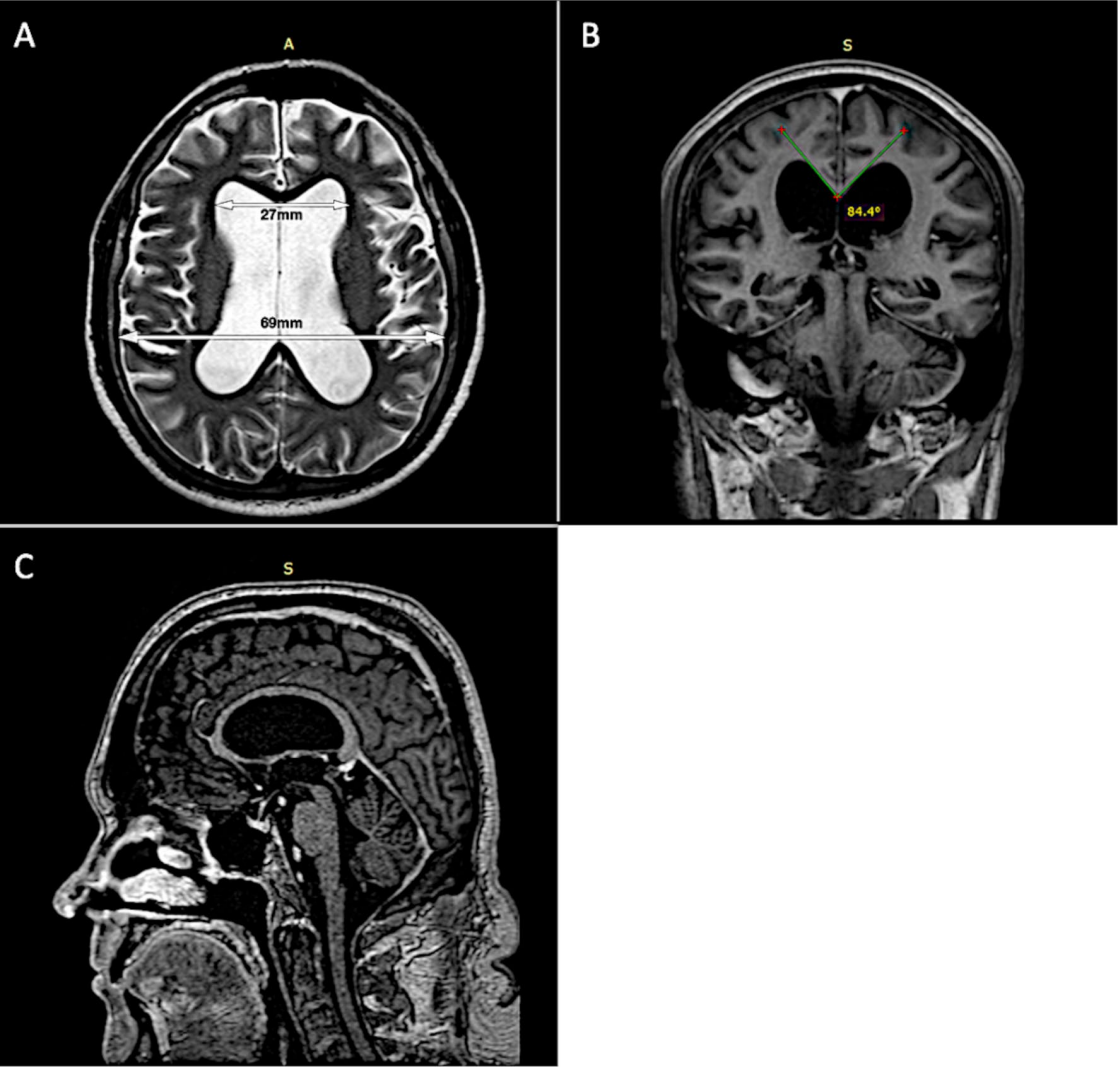
Brain MRI in a patient with normal pressure hydrocephalus after COVID19 infection showing: **A** Axial T2-weighted image with dilated lateral ventricles, **B** coronal T1-weighted image depicting acute callosal angle and upward bowing of the corpus callosum and **C** sagittal T1-weighted image showing normal infratentorial ventricular compartment

Brain magnetic resonance imaging (MRI) disclosed moderately dilated supratentorial ventricular system with callosal angle reduction and aqueduct flow-void (Fig. 1). A high-volume (40 ml) lumbar tap test was performed and revealed an opening pressure of 100mmH2O (normal range < 200 mmH_2_O). There was marked improvement of gait and balance after the procedure—10 m timed up and go (TUG) before the procedure of 18.4 s (32 steps) and after the procedure of 16.7 s (27 steps). CSF analysis was entirely normal (Table 1).

A diagnosis of Normal Pressure Hydrocephalus was made and a ventriculoperitoneal shunt was placed without adverse events. Thirty days later there was significant improvement of gait, now presenting normal speed and step length. Cognitive functions also improved substantially, and he returned to his previous work activities. The patient reports that his ability to perform his labor activities is normal and is very pleased with his clinical outcome.

## Discussion and conclusion

We have described a previously healthy patient who developed NPH two months after COVID19 infection, presenting with gait apraxia and cognitive impairment.

Normal pressure hydrocephalus has been classified as idiopathic or secondary [3]. Many diseases have been implicated in its etiology, such as subarachnoid hemorrhage (SAH), traumatic brain injury (TBI), meningitis, stroke and intracranial neoplasms [3]. Some specific infectious diseases have also been reported as possible etiologies for NPH, including Lyme disease, neurosyphilis and cryptococcosis [4]. There are reports of hydrocephalus following SAH in patients with concomitant coronavirus infections [5, 6], but as far as we know this is the first case of NPH associated with COVID-19 infection without preceding SAH or stroke.

The main theory to explain central nervous system (CNS) invasion is that SARS-CoV-2 probably enters olfactory nerves through interaction of the viral S protein with the Angiotensin Converting Enzyme 2 (ACE2) receptor on olfactory epithelium [1]. The spatial distribution of ACE2 receptors in the CNS was studied using brain transcriptome data banks and high concentrations of these receptors were found in the choroid plexus of lateral ventricles suggesting that the choroid plexus could be a pathway for SARS-CoV-2 to enter the CNS [7]. It is possible that this interaction between the virus and the choroid plexus could alter the dynamics of CSF flow, contributing to NPH. An animal model study of hydrocephalus after SAH has demonstrated that inflammation of choroid plexus cells marked by increase in NF-kB results in dysfunction of these barrier cells leading to production of increased abnormal protein-rich CSF, and consequentially in hydrocephalus due to excess CSF production [8].

Many neuroinvasive viruses such influenza A and B can initially trigger an innate immune response by activating microglia [9]. Microglia also activate astrocytes that can modulate the recruitment and activation of additional microglial and other immunocompetent cells [10]. Microglial and astrocyte activation as well as massive cytokine release might lead to inflammation of arachnoid villi resulting in fibrosis and adhesion of these structures, reduction in CSF reabsorption, and consequently to hydrocephalus [11]. Another possible factor in the etiology of COVID19-associated NPH is a hypercoagulable state induced by the systemic response to SARS-CoV-2 [12], which could lead to venous congestion and CSF flow abnormalities [11].

In a series of patients with persistent headache after COVID-19 infection 84.6% had increased intracranial pressure (ICP) in the absence of meningitis [13]. It is possible that some of these patients with initially elevated ICP might progress to NPH over time as the CSF compartment rearranges itself in a larger volume to maintain physiologic ICP according to Laplace’s law [14].

Unfortunately, we were not able to test CSF for COVID antibodies due to local technical limitations. In a recent study of 8 patients with COVID-associated encephalopathy (ref [1]) all patients had positive anti-SARS-CoV-2 antibodies in CSF, but only 4 of them (50%) had high titers comparable to blood titers. Among these 4 patients, only 1 had intrathecal anti-SARS-CoV-2 IgG synthesis, the remainder had disruption of the blood–brain-barrier. As such, it is still unclear whether specific anti-SARS-CoV-2 antibodies play a role in neurologic manifestations associated with COVID, or if blood–brain-barrier dysfunction with cytokine influx is the main component in such manifestations [15].

We have described the first case of normal pressure hydrocephalus possibly associated with COVID19 infection. Although pathophysiological mechanisms are still unclear and we cannot be certain that NPH occurred because of COVID19, there was a clear temporal correlation, and the patient did not have other risk factors for NPH. Given the significance of this condition as a cause of reversible dementia, it is important to consider the possibility of a causal association with COVID19. More reports are necessary to confirm this association and clarify its mechanisms.

## Data Availability

All data generated or analysed during this study are included in this published article.

## Abbreviations

ACE2: Angiotensin converting enzyme 2
CNS: Central nervous system
ICP: Intracranial pressure
MRI: Magnetic resonance imaging
NPH: Normal pressure hydrocephalus
SAH: Subarachnoid hemorrhage
TBI: Traumatic brain injury
